# Persian Traditional Medicine and Ocular Health

**Published:** 2015

**Authors:** Hasan Namdar, Elham Emaratkar, Mohammad Bagher Hadavand

**Affiliations:** Department of Traditional Medicine, School of Medicine, Shahed University, Tehran, Iran

**Keywords:** Persian Traditional Medicine, Ocular Health, Vision

## Abstract

The Persian Traditional Medicine (PTM) system pays special attention to disease prevention. In PTM, physicians believe that overeating may cause accumulation of unhealthy substances in the body and diseases called “Emtela.” With respect to ocular health, foods can be categorized as beneficial and harmful. Harmful foods such as beef, geese, eggplant, cauliflower, and cheese can cause reduced vision. Dehydrating foods such as walnut and salty fish and hot foods such as garlic, onion, and pepper can cause dry eye. Food items that have beneficial effects on ocular health include thyme and saffron and fruits such as grape, fig, apple, plum, and berries. PTM stipulates that one should not drink water with meals or immediately afterwards, since drinking cold (icy) water causes difficulty in absorption of nutrients. Gulping water may have harmful effects on the eyes; therefore, PTM physicians recommend drinking water at a suitable temperature. It is not safe to drink water first at the morning. Sleeping right after eating is harmful too. Avicenna believes that sleeping on one’s belly after a full meal is very harmful for the eyes. Galen says that old people need deep and continuous sleep more than others. From the view of PTM, moving eyes in different directions, making delicate expressions, trying to look at delicate and find pictures and reading small letters would remove ocular fatigue. There have been mentions of local medicine for improving vision as well; for instance, fennel extracts, pomegranate juice, and honey which are suitable for vision improvement. Local administration of pomegranate blossoms is suitable for treating inflammatory reactions.

## INTRODUCTION

The modern medicine emphasis on different aspects of of preventive medicine (1). In Persian Traditional Medicine (PTM), special attention is paid to preventing diseases and in the resources of this medical scheme, taking necessary measures to keep organs healthy prior seeking medical treatment (2, 3).

From the viewpoint of PTM, the human body is composed of four groups of substances, which are called the four humors (Akhlat): yellow bile (Safra humor), blood humor (Dam), phlegm humor (Balgham), and black bile humor (Sauda) (2,4-6, 8). Depending on the quantity of each humor, the human body shows a dominant quality of one of the four humors, i.e., hot cold, dry, and moist, which are called temperaments (9). Changes in the quantity or quality of each of the humors would cause unnatural changes in the four qualities, which are called “sue mizaj” (5, 10-12). Sue mizaj (ill temperament) is a kind of disease in traditional medicine and could cause disorders in natural activities (2,6,8). The increase in dry eye (dry sue mizaj) caused by consumption of foods such as walnut or pepper leads to itching and a burning sensation in the eye and/or coldness of eye tissues (cold -sue mizaj), which causes an increase in tearing (13-14). Maintenance of eye health requires good health in other parts of the body, especially the brain. This is because eyes are affected by the brain and other parts of the body (15), while an increase in the levels of some substances in the brain could cause ocular disease (16-17). This manuscript discusses the general principles of traditional medicine related to improving vision and preventing ocular diseases in view of PTM.

## METHOD

In this review, manuscripts related to “ophthalmology and PTM” published from 1995 to September 2015 were searched in Pubmed and Scopus. By using the comprehensive library of traditional medicine of Shahed University, Tehran, Iran, University of Tehran and the software used by Noor Digital Library, Tehran, Iran, manuscripts and related publications such as Al-Qanon fi al-Tibb, Noor al-Oyoun, Gameo al- Fonoun were searched carefully. General traditional Persian medical books that discussed ophthalmology, such as Al-Qanon fi al-Tibb and Zakhireh Kharazmshahi, and Qanoun of Avicenna were also studied. 

## RESULTS

Although during Islamic civilization, ophthalmology was relied on Greek resources, it has shown significant progress in the lapse of time. Some specialized and independent books on ophthalmology were written in this era. These books contained valuable recommendations to maintain ocular health. The recommendations generally focus on foods, beverages, medicine, activities, sleep, removal of extra substances from the body, and stability of the mental states (2, 17).

**1- Foods**

Physicians of PTM believed that if a person overeats or eats foods before full digestion of the last meal, the frequency of this practice could cause accumulation of substances in the body and diseases called Emtela (2,17). The foods discussed in connection with eyes are divided into beneficial and harmful substances.

*•Foodstuffs that harm eyes*

A- Concentrated foods such as beef, geese, eggplant, cauliflower, and cheese (7,18) that produce concentrated blood and slow blood flow in body organs are harmful for ocular health (17). In addition, digestion of concentrated foods is more difficult and is associated with indigestion; therefore, they cause accumulation of substances in the body and consequent impairments in vision (7, 19).

B- Dried foods such as walnut, salted fish, and saline cause dryness in body tissues including the eye, and may cause poor vision in the future (17).

C- Flatulence and vapor-causing foods such as peas, beans, lentils, vegetables, and horseradish cause excessive gas and steam that are harmful for eyes (17).

D- Garlic, onion, and pepper may cause dryness in the body and harm the eyes (2,17). 

*•Beneficial foods*

Thyme and saffron are among the foods that may improve ocular health (2,5). In addition, foods such as grape, fig, apple, plum, and berry are useful for the eye. PTM physicians recommended that after intense physical activity, moisture-containing fruits such as apple, grape, and fig must be taken to increase body moisture levels and compensate for the dryness caused by the exercises (17).

**2- Beverages**

Drinking water is necessary for digestion, absorption, and distribution of substances; therefore, according to Avicenna, the body requires water for processing food in the stomach (2,17). To prevent ocular diseases, one must pay attention to the following recommendations:

A. It is not recommended to drink water with food and immediately after a meal because it causes disorders in food digestion and subsequently leads to several diseases

B. Drinking icy water is harmful since it may affect digestion.

C. It is not recommended to drink water immediately after strenuous physical activity because this causes severe weakness of various organs, including the eyes.

D. Gulping water is harmful for the eyes because it rapidly reduces the temperature of the internal organs and leads to weakness of the organs, including the eye (2,12, 17).

**3. Sleep**

In PTM, proper sleep is recommended. Adequate sleep causes improvement of the body and various organs. Sleeping immediately after eating food and with a full stomach is harmful for different organs, including the eye (2,11). Avicenna believed that sleeping with a full stomach is the most harmful habit. Proper sleep is a sleep which is suitable and balanced in terms of duration. Oversleeping and insufficient sleep are both harmful for ocular health. Oversleeping causes concentration of substances in the eye tissues and poor vision (2). Staying awake for a long time diminishes the levels of substances in the eye and harms the eye by causing dry eye (12,17). Galion believed that old people need deep and suitable sleep more than others; therefore, these individuals should use sleep-improving substances such as lettuce every night in order to have appropriate sleep (17). 

**4. Physical activities**

According to PTM, physical activity reinforces digestion, opens the pores inside the body, discharges additional substances, causes tissue reinforcement, and thus increases the body’s ability to perform its functions (2, 17). Movement of eye in different positions, performing delicate work, looking at fine and delicate objects and pictures, reading small letters, and gazing to a point are considered ocular muscle activities (2, 17). Specialized activities exercising each organ will cause better absorption and discharge of substances from the organ and prevent fatigue. In contrast, lack of exercise would cause closure of the body’s pores, accumulation of substances, and the feeling of fatigue (12).

**5. Cleaning**

PTM physicians believe that accumulation of excessive amounts of substances in the body causes poor vision (12). Urination, excretion, perspiration, and vomiting are the processes that the body uses to clean itself. The health of organs, including the eye, is maintained when the discharge systems maintain the internal balance of body by discharging excessive substances (17).

In PTM, vomiting can sometimes improve vision. However, too much vomiting causes dryness and is harmful for the eyes. In addition, if vomiting is accompanied by intense movement, it might cause harm to the eye and ocular hemorrhage (12). 

**6. Taking baths**

Baths usually cause an increase in the heat and moisture in body organs. Extended baths can cause excessive perspiration and dehydration, including dry eye and poor vision. Individuals who use a sauna for losing weight suffer from dehydration due to too much perspiration and show impaired vision. PTM believe that taking a bath at the beginning of the day in the morning and in hunger causes thinness and is harmful for vision (17). Dipping one’s head in cold and clean water and opening eyes under water causes ocular refreshment (2,17). From the viewpoint of PTM, using cold water following exercise may be useful in freshness and improve vision (17).

**7. Psychological factors**

PTM believes in the effect of psychological factors on substances in the body. While mental conditions such as depression and anxiety cause changes in the quality and quantity of substances in the body, the same conditions also cause changes in substances inside the body (17,20). Too much sorrow, crying, and stress are factors that physicians believe to be the cause of dryness and dehydration of the body and eyes (2,17). Joy and happiness can improve vision. Enjoyment improves body strength and improves the five senses, especially vision (17).

**8. Medications**

In PTM, other dissolved local medicines are recommended for improving vision such as fennel juice, pomegranate juice, and honey, which are prepared using a special method and are useful for improving vision (2, 6, 19). Local administration of pomegranate blossoms is suitable for treating inflammatory reactions of the eye (17). Local use of saffron or consumption of saffron Sherbet prevents various ocular diseases and improves vision (5). The climate conditions are also effective in the ocular health. Dust, cold and too much heat are harmful for the eye and cause irritation and burn of eye (2).

## Discussion

The importance of protecting eyes and preventing ocular disorders and complications is evident from the role of eyes in people’s lives. The recommendations of PTM for preventing ocular diseases can be grouped with respect to the mechanism associated with the harmful effects on eyes:

1. Dry eye: Some of these factors directly cause dry eye and if they continue, they might cause dehydration in the body as well; these include gazing in a fire for a long time, too much crying, or gazing at bright objects. However, some factors cause dry eye following dehydration of body. These factors include edible substances such as salty and hot foods, lack of sleep, too much sexual activity, too much perspiration, and repeated vomiting.

2. Factors that cause flatulence and gas: Peas, beans, vegetables and horseradish, and drinking cold water with foods cause excessive vapor inside the body, including eye tissues, and impair the natural functions of the eye.

3. Factors that cause Emtela: Lack of physical activity, insufficient exercise, incomplete digestion of food, overeating, or eating foods before digestion of the previous meal can cause accumulation of wastes in the eyes and result in ocular fatigue and poor vision.

4. Concentrating factors: Oversleeping or eating beef can cause concentration of moisture inside the body. These factors slow down blood flow inside the ocular tissue canals and are followed by poor vision.

In conclusion, we recommend consideration of these valuable inputs for preventing ocular diseases after clinical trials.

## References

[1] Hadavand MB, Heidary F, Heidary R, Gharebaghi R (2013). Role of ophthalmic nurses in prevention of ophthalmic diseases. Med Hypothesis Discov Innov Ophthalmol.

[2] Avicenna Hossein Bin Abdullah (2005). Al-Qanon fi al-TibbQanun Fe Tebb, 10th and 11th century, research by Shamseddin Ebrahim.

[3] Gorgani Seyed Esmaeil (1979). Zakhireh Kharazmshahi.

[4] Amoli Shamseddin Mohammad Bin Mahmoud (2005). Comments on Ghanoun Amoli, Manuscript.

[5] Guilani Ali Comments on Ghanoun Amoli, Manuscript, Islamic Consultative Assembly Library.

[6] Gooshah Gir AA, Namdar H, Emaratkar E, Nazem E, Minaii MB, Nikbakht Nasrabadi AR, Choopani R (2013). Avicenna's view on the prevention of thrombosis. Int J Cardiol.

[7] Ghorashi Ebn Nafis Ali Bin Abelhazm (2003). Comments on Qanoun.

[8] Choopani R, Mosaddegh M, Gir AA, Emtiazy M (2012). Avicenna (Ibn Sina) aspect of atherosclerosis. Int J Cardiol.

[9] Ahvazi Ali Kamel Alsenat Altayebeh (2008). Correction and Research, Restoration of Natural Medicine Institute, ordered by the Institute of Medical History, Islamic Medicine.

[10] Ebn Nafis Ali Ben Abialhazm (2001). Almujez Fe Tebb, research by Abdolkarim Alqarbavi.

[11] Ebn Hendu (2008). Mafateh Alteb.

[12] Hariri Abdollah Bin Qasem (1979). Nahayatollah Afkar and Nezhatol Absar.

[13] Arzani Mohammad Akbar (2008). Akbari Medicine, Correction and Research, Institute of Natural Medicine, ordered by the Institute of Medical History, Islamic and Supplementary Medicine.

[14] Viirre E (2014). Dynamic assessment of binocular eye movement coordination: norms and functional implications. Med Hypothesis Discov Innov Ophthalmol.

[15] Alkahal AliBin Issa (2008). Tazkoratol Alkahalin.

[16] Georgani Seyed Esmaeil (2005). Alaghraz Altayebeh val Mabahesol Alanieh, Correction and Resarch by Dr. Hassan Tajbakhsh.

[17] Hemavi Salaheddin Ben Yousef Kahal (1986). Noor al- 0youn, Gameo al- FonounNourol Oyoun and Jameol Fonoun.

[18] Aghili Khorasani, Seyed Mohammad Hossein (2008). Makhzanol Advieh.

[19] Hakim Shamseddin Ahmad Khazaenol Moluk (2002). 18-19th century.

[20] Shirazi Ghotbeddin (2001). Tohfatol Sadieh, Manuscripts of Islamic Legislative Assembly Library.

